# White matter alterations in Alzheimer’s disease without concomitant pathologies

**DOI:** 10.1111/nan.12618

**Published:** 2020-05-01

**Authors:** I. Ferrer, P. Andrés‐Benito

**Affiliations:** ^1^ Department of Pathology and Experimental Therapeutics University of Barcelona Barcelona Spain; ^2^ Bellvitge University Hospital Barcelona Spain; ^3^ Ministry of Economy and Competitiveness CIBERNED (Network Centre of Biomedical Research of Neurodegenerative Diseases) Institute of Health Carlos III Barcelona Spain; ^4^ Institute of Neurosciences University of Barcelona Barcelona Spain; ^5^ Bellvitge Biomedical Research Institute (IDIBELL) Barcelona Spain

**Keywords:** Alzheimer disease, co‐morbidities, myelin, oligodendrocytes, white matter

## Abstract

**Aims:**

Most individuals with AD neuropathological changes have co‐morbidities which have an impact on the integrity of the WM. This study analyses oligodendrocyte and myelin markers in the frontal WM in a series of AD cases without clinical or pathological co‐morbidities.

**Methods:**

From a consecutive autopsy series, 206 cases had neuropathological changes of AD; among them, only 33 were AD without co‐morbidities. WM alterations were first evaluated in coronal sections of the frontal lobe in every case. Then, RT‐qPCR and immunohistochemistry were carried out in the frontal WM of AD cases without co‐morbidities to analyse the expression of selected oligodendrocyte and myelin markers.

**Results:**

WM demyelination was more marked in AD with co‐morbidities when compared with AD cases without co‐morbidities. Regarding the later, mRNA expression levels of *MBP*, *PLP1*, *CNP*, *MAG*, *MAL*, *MOG* and *MOBP* were preserved at stages I–II/0–A when compared with middle‐aged (MA) individuals, but significantly decreased at stages III–IV/0–C. This was accompanied by reduced expression of *NG2* and *PDGFRA* mRNA, reduced numbers of NG2‐, Olig2‐ and HDAC2‐immunoreactive cells and reduced glucose transporter immunoreactivity. Partial recovery of some of these markers occurred at stages V–VI/B–C.

**Conclusions:**

The present observations demonstrate that co‐morbidities have an impact on WM integrity in the elderly and in AD, and that early alterations in oligodendrocytes and transcription of genes linked to myelin proteins in WM occur in AD cases without co‐morbidities. These are followed by partial recovery attempts at advanced stages. These observations suggest that oligodendrocytopathy is part of AD.

## Introduction

Reduced white matter (WM) volume, WM lesions and altered WM integrity and cortical disconnection occur in the ageing human brain [1, 2, 3, 4, 5, 6, 7, 8, 9, 10]. Age‐related WM decay is associated with memory impairment and symptoms of depression in an anatomically specific manner [1, 2, 3, 4, 5, 6, 7, 8, 9, 10, 11, 12, 13, 14]. Reduced myelin basic protein (MBP) and 2',3'‐cyclic nucleotide 3' phosphodiesterase (CNP) levels [15], and alterations in the number of oligodendrocytes and oligodendroglial precursor cells (OPCs/NG2‐positive cells), have been reported in aged primates and rodents [16].

Reduced WM size, WM hyper‐lucencies and myelin and axon damage are common in sporadic Alzheimer’s disease (AD), as revealed by neuroimaging methods, mainly magnetic resonance imaging (MRI), and particularly diffusion tensor imaging (DTI) and functional MRI (fMRI) [3, 4, 12, 14, 17, 18, 19, 20, 21, 22]. Patients with moderate cognitive impairment (MCI) of AD‐type exhibit alteration of the WM integrity which further deteriorates with disease progression [5, 23, 24, 25, 26, 27]. WM alterations appear before the appearance of clinical symptoms [28]. Atrophy of WM, decreased myelin density and demyelination and predominant WM vulnerability of the frontal and parietal lobes are also observed in *post mortem* neuropathological studies [2, 29, 30, 31, 32]. Myelin loss mainly involves areas that are myelinated late in the development [8, 33, 34]. Breakdown of WM integrity is considered a principal component of AD, contributing to neural disconnection and progression of clinical symptoms and dementia [13].

However, most studies of WM in the human ageing brain have not considered that in the elderly, most individuals have AD pathology that cannot be visualized with current neuroimaging methods. Therefore, we cannot be certain that a reasonable percentage of aged individuals subjected to neuroimaging studies suffer concomitant AD pathology. Moreover, the presence of a certain number of β‐amyloid plaques and neurofibrillary tangles (NFTs) in the elderly have been considered until recently to be normal brain ageing; these cases might have been classified at *post mortem* as normal aged individuals.

Furthermore, co‐morbidities are common in the ageing brain and in AD. Vascular cognitive impairment and dementia are prevalent in old age [35, 36, 37, 38, 39, 40, 41, 42]. The combination of AD and cerebrovascular disease is very common [43, 44, 45, 46, 47, 48, 49]. Other clinical co‐morbidities include arterial hypertension (HTA), type II diabetes, cardiac, hepatic and renal failure and respiratory insufficiency; pathological co‐morbidities include neurodegenerative disorders with abnormal protein aggregates such as other tauopathies, Lewy body diseases and TDP‐43 proteinopathies, among others and hippocampal sclerosis [50, 51, 52, 53, 54, 55, 56].

This study was designed to analyse molecular alterations in the WM linked to oligodendrocytes and myelin in cases with AD pathological changes without clinical or pathological co‐morbidities. These cases were first selected from a consecutive series of autopsies in a general hospital in which AD was one of the *post mortem* neuropathological diagnoses. Cases with clinical and pathological co‐morbidities were not included in the second part of the study.

In the second part of the study, gene transcription was assessed with targeted RT‐qPCR, and protein expression of altered genes by immunohistochemistry in the WM of the centrum semi‐ovale of the frontal lobe in cases with AD pathology without clinical and pathological co‐morbidities at different Braak stages of disease progression. Selected genes for analysis included markers of the oligodendrocyte lineage, genes encoding structural proteins of myelin and genes involved in energy metabolism and axon maintenance. Adult NG2‐glia have the capacity to produce myelinating oligodendrocytes [57, 58] thus contributing to oligodendrocyte and myelin turnover and regeneration in the adult CNS [59]. Stages of oligodendrocyte lineage are identified by the expression of platelet‐derived growth factor receptor α polypeptide (PDGF‐Rα), SRY‐Box‐10 (Sox10), NK2 homeobox 2 (Nkx2.2), oligodendrocyte transcription factor 1 (Olig1) and oligodendrocyte lineage transcription factor 2 (Olig2), among others [60]. After oligodendrocyte differentiation, myelination is triggered by myelin regulatory factor (MYRF) which is expressed in postmitotic oligodendrocytes [61]. Myelination is linked to increased expression of myelin basic protein (MBP), myelin‐associated glycoprotein (MAG), proteolipid protein 1 (PLP1), myelin oligodendrocyte glycoprotein (MOG) and 2′, 3′‐cyclic nucleotide 3′ phosphodiesterase (CNP), among others [62, 63, 64, 65, 66, 67, 68]. Finally, blood‐derived glucose is taken up by oligodendrocytes through glucose transporter 1 (GLUT1) encoded by *SLC2A1*. Glucose is metabolized via glycolysis to produce pyruvate and lactate which are delivered to the axons through specific solute carriers, the monocarboxylate transporters (MCT) located in cell membranes [69, 70]. MCT1 is mainly expressed in oligodendrocytes [71, 72]. Thus, both GLUT1 and MCT1 produced in oligodendrocytes are involved in axon maintenance independently of the complementary myelin/axon trophic alliance. These markers were analysed in this study.

Finally, histone deacetylases (HDACs) remove specific acetyl groups on a histone enabling it to interact with DNA thereby modulating gene transcription. HDAC1 and HDAC2 are expressed in oligodendrocytes [73, 74]. HDAC1 immunoreactivity was also assessed in the nuclei of glial cells in the WM.

Our aim was to discern which alterations in the WM in the general population affected by AD pathological changes were linked to AD and not to concomitant clinical and pathological co‐morbidities.

## Material and methods

### Selection of samples

#### First series

Cases for study were obtained at the Bellvitge University Hospital following the guidelines of the Spanish legislation (Real Decreto 1716/2011) and the approval of the local ethics committee. Clinical parameters were retrieved after the revision of the complete clinical history in every case. Only cases with comprehensive clinical information were separated for further analysis. The first series of cases was chosen from consecutive autopsies carried out from 2009 to 2015. The current protocol for the autopsies in adult donors was as follows: one hemisphere was immediately cut in coronal sections, 1 cm thick and selected areas of the encephalon were rapidly dissected, frozen on metal plates over dry‐ice, placed in individual air‐tight plastic bags and stored at −80°C until use for biochemical studies. The other hemisphere was fixed by immersion in 4% buffered formalin for 3 weeks for morphological studies. For the current neuropathological study, 4‐µm‐thick sections from 20 representative brain regions were stained with haematoxylin and eosin, periodic acid‐Schiff (PAS) and Klüver–Barrera, or processed for immunohistochemistry for microglia Iba1, glial fibrillary acidic protein (GFAP), β‐amyloid, phospho‐tau (clone AT8), α‐synuclein, TDP‐43 and ubiquitin, using EnVision + System peroxidase (Dako), and diaminobenzidine and H_2_O_2_. In addition, 1‐cm‐thick coronal sections of the frontal lobe at the level of the head of the caudate and putamen were obtained in every case. Blocks were embedded in paraffin, cut at a thickness of 7 µm, de‐waxed and stained with haematoxylin and eosin, and with Klüver–Barrera. Details of the 20 selected regions and the methodological protocols for current neuropathological studies are described elsewhere [75]. From among autopsies following this procedure, including the availability of appropriate clinical information and suitability of white matter sections stained with Klüver–Barrera for densitometric studies, 470 of 713 autopsies were initially selected. Three groups were defined. First, all cases with NFT pathology and/or with cerebral β‐amyloid deposition, covering NFT pathology stages I–VI of Braak and β‐amyloid deposition in the form of diffuse and/or neuritic plaques Braak stages 0 (no deposits) to C [76], were chosen for further classification. These were 128 men and 78 women (total *n* = 206); mean age (±SEM): 73.3 ± 9.6 years. AD cases were categorized as ADI–II/0‐A (*n* = 113, men: 83, women: 30; age: 69.1 ± 8.9 years); ADIII–IV/0–C (*n* = 70, men: 36, women: 34; age: 78.4 ± 7.6 years) and ADV–VI/B–C (*n* = 23, men: 9, women: 14; age: 78.5 ± 8.5 years). Most of these cases encompassed a diversity of co‐morbidities including metabolic disorders such as HTA, type II diabetes, hyperlipaemia, renal or liver failure and chronic respiratory failure; peripheral and central vascular pathology; age‐related neurodegenerative diseases, such as other tauopathies, Lewy body pathology and TDP‐43 proteinopathy, among others; hippocampal sclerosis and long agonic state, or vegetative state. Regarding cerebrovascular pathology, cases with cerebral infarcts, including micro‐infarcts, lacunes, infarcts in the watershed areas, vascular leucoencephalopathy, hippocampal sclerosis and status cribosus; and cases with severe atherosclerosis, moderate or severe artheriolosclerosis, hypertensive angiopathy, inflammatory vascular diseases and vascular malformations were considered as AD with vascular co‐morbidity. This group was classified as AD with concomitant pathology, AD‐Co, and was made up of 173 cases, categorized as ADI–II/0–A (*n* = 104), ADIII–IV/0–C (*n* = 63) and ADV–VI/B–C (*n* = 7).

Only 33 of the 206 were classified as cases with AD without co‐morbidities. Cases with concomitant mild small blood vessel disease were included in this series. This group was made up of by 15 men and 18 women; age: 76.3 ± 8.6 years. AD cases without co‐morbidities were categorized as follows: ADI–II/0–A (*n* = 9, men: 5, women: 4; age: 68.5 ± 11.3 years); ADIII–IV/0–C (*n* = 8, men: 2, women: 6; age: 77.7 ± 4.8 years); ADV–VI/B–C (*n* = 16, men: 8, women: 8; age: 80.7 ± 5.6 years). All these cases were sporadic; familial AD was not included in this study.

The third group (*n* = 20) was made up of young cases (middle‐aged: MA) chosen at random among the remaining 264 cases (470 minus 206). This group consisted of 10 men and 10 women; age: 48.1 ± 7.7 years. MA did not have the clinical risk factors and co‐morbidities mentioned in previous paragraphs; they did not have neurological or mental diseases, and the neuropathological study did not show abnormalities. The control group must be not interpreted as an age‐matched control group, but as a control group of normal WM in MA individuals.

#### Second series

Only cases with AD pathology without co‐morbidities were the focus of this part of the study. These cases were the same as those of the second group of the first series. In addition, 10 MA cases (6 men, 4 women; age: 51.7 ± 4.7 years) from the third group of the first series were included and processed in parallel. The total number of MA and AD cases in this series is detailed in Table 1. All these cases were used for specific immunohistochemical studies. However, frozen samples were available for 24 AD cases and 10 controls. In these cases, the brain pH at the time of the autopsy was between 6.2 and 6.4, and the RNA integrity number (RIN) higher than 6 (excepting one case). AD cases at stages I–II/0‐A had no neurological symptoms; AD cases at stages III–IV/0–C had no neurological symptoms nor were they affected by mild cognitive impairment; AD cases at stages V–VI/B–C had severe cognitive impairment or dementia.

**Table 1:** Summary of cases of AD without concomitant co‐morbidities and pathologies

Case ID	Diagnosis	Sex	Age	PM delay	RIN WM
1	AD I/0	M	56	07 h 10 min	7.80
2	AD I/A	W	74	02 h 45 min	7.70
3	AD I/A	W	57	05 h 00 min	6.50
4	AD I/A	M	66	09 h 45 min	6.30
5	AD II/0	M	67	07 h 15 min	6.90
6	AD II/0	M	57	04 h 30 min	7.10
7	AD II/A	W	88	08 h 00 min	6.90
8	AD II/A	M	66	04 h 55 min	7.50
9	AD II/A	W	86	02 h 15 min	8.30
10	AD III/0	M	81	01 h 30 min	7.60
11	AD III/0	M	66	05 h 45 min	7.50
12	AD III/0	W	79	03 h 35 min	7.40
13	AD III/A	W	82	02 h 00 min	7.20
14	AD III/A	W	77	03 h 10 min	6.40
15	AD III/B	W	76	03 h 50 min	7.20
16	AD IV/A	W	80	02 h 45 min	5.40
17	AD IV/C	W	81	05 h 00 min	7.30
18	AD V/B	W	74	05 h 30 min	7.70
19	AD V/B	M	86	04 h 15 min	7.90
20	AD V/B	M	73	04 h 30 min	6.90
21	AD V/B	W	82	01 h 45 min	–
22	AD V/B	M	75	11 h 30 min	–
23	AD VI/C	W	72	09 h 30 min	6.40
24	AD VI/C	W	85	16 h 15 min	8.20
25	AD V/C	M	85	03 h 45 min	7.80
26	AD V/C	M	77	16 h 00 min	7.10
27	AD VI/C	W	82	10 h 00 min	–
28	AD V/C	W	86	10 h 00 min	–
29	AD V/C	W	85	12 h 10 min	–
30	AD V/C	M	77	08 h 00 min	–
31	AD V/C	M	79	07 h 30 min	–
32	AD V/C	M	93	03 h 00 min	–
33	AD VI/C	W	81	05 h 15 min	–
34	MA	W	62	11 h 00 min	8.40
35	MA	W	53	03 h 00 min	7.70
36	MA	M	55	05 h 40 min	8.30
37	MA	M	39	09 h 15 min	7.10
38	MA	W	46	14 h 05 min	7.20
39	MA	W	66	04 h 15 min	7.80
40	MA	M	57	03 h 00 min	8.20
41	MA	M	50	17 h 15 min	8.00
42	MA	W	54	06 h 45 min	7.20
43	MA	M	35	17 h 00 min	6.80

### Quantitative densitometric studies of the WM stained with Klüver–Barrera

Photomicrographs of the centrum semi‐ovale of Klüver–Barrera‐stained sections, at the level of the head of the caudate and putamen as described above, were obtained at a mid‐distance between the inferior frontal sulcus and the cingulate sulcus, approximately at 0.7–1 cm from the external vertex of the lateral ventricle. Figures were obtained at a magnification of × 200, covering an area of 0.126 mm^2^, using a DP25 camera adapted to an Olympus BX50 light microscope_._ The pictures, two areas per case in every case, were analysed using Photoshop software. The density of myelin was calculated as the intensity of blue normalized for the total area and expressed as arbitrary units per area. The normality of distribution was analysed with the Kolmogorov–Smirnov test. Results were analysed with one‐way anova and post hoc Tukey. Differences between MA and the two groups of AD, and between the different stages in pure AD cases, were considered statistically significant at *P < 0.05, **P < 0.01, ***P < 0.001 when comparing MA with AD and AD‐Co, and ^##^ *P* < 0.01 when comparing AD‐Co with ‘pure’ AD.

### RNA purification and RT‐qPCR

The WM was dissected from the grey matter in 34 cases, corresponding to 9 ADI–II/0–A, 8 ADIII–IV/0–C, 7 ADV–VI/B–C and 10 MA. WM samples at different AD stages and MA cases were processed in parallel. RNA from frozen WM was extracted following the instructions of the supplier (RNeasy Mini Kit, Qiagen^®^ GmbH, Hilden, Germany). RNA integrity and 28S/18S ratios were determined with the Agilent Bioanalyzer (Agilent Technologies Inc, Santa Clara, CA, USA). RIN values are shown in Table 1. Samples were treated with DNase digestion, and RNA concentration was evaluated using a NanoDrop™ Spectrophotometer (Thermo Fisher Scientific, Waltham, MA, USA).

TaqMan RT‐qPCR assays were performed in duplicate for each gene on cDNA samples in 384‐well optical plates using an ABI Prism 7900 Sequence Detection system (Applied Biosystems, Life Technologies, Waltham, MA, USA). For each 10 μl TaqMan reaction, 4.5 μl cDNA was mixed with 0.5 μl 20 × TaqMan Gene Expression Assays and 5 μl of 2 × TaqMan Universal PCR Master Mix (Applied Biosystems). TaqMan probes used in the study are detailed in Table 2. Values of *GUS‐β* were used as internal controls for normalization [77]. The parameters of the reactions were 50°C for 2 min, 95°C for 10 min and 40 cycles of 95°C for 15 s and 60°C for 1 min. Finally, capture of all TaqMan PCR data was made using the Sequence Detection Software (SDS version 2.2.2, Applied Biosystems). For the data analysis, threshold cycle (CT) values for each sample were processed to obtain the double delta CT (ΔΔCT) values. First, delta CT (ΔCT) values were calculated as the normalized CT values for each target gene in relation to the CT of endogenous controls *GUS‐β*. Then, ΔΔCT values were obtained from the ΔCT of each sample minus the mean ΔCT of the population of control samples.

**Table 2:** Taqman probes, gene names and identification

Gene	Full name	Reference
*CNP*	2',3'‐cyclic nucleotide 3' phosphodiesterase	Hs00263981_m1
*GUS‐β*	β‐glucuronidase	Hs00939627_m1
*MAG*	Myelin‐associated glycoprotein	Hs01114387_m1
*MAL*	Mal, T‐cell differentiation protein	Hs00360838_m1
*MBP*	Myelin basic protein	Hs00921945_m1
*MCT1*	Solute carrier family 16 (monocarboxylic acid transporters), member 1	Hs01560299_m1
*MOBP*	Myelin‐associated oligodendrocyte basic protein	Hs01094434_m1
*MOG*	Myelin oligodendrocyte glycoprotein	Hs01555268_m1
*MYRF*	Myelin regulatory factor	Hs00973739_m1
*NG2*	Neural/glial antigen 2	Hs00426981_m1
*NKX2‐2*	NK2 Homeobox 2	Hs00159616_m1
*OLIG1*	Oligodendrocyte transcription factor 1	Hs00744293_s1
*OLIG2*	Oligodendrocyte lineage transcription factor 2	Hs00377820_m1
*PDGFRA*	Platelet‐derived growth factor receptor, alpha polypeptide	Hs00998018_m1
*PLP1*	Proteolipid protein 1	Hs00166914_m1
*SLC2A1*	Solute carrier family 2 (facilitated glucose transporter), member 1	Hs01102423_m1
*SOX‐10*	SRY‐box 10	Hs00366918_m1

The normality of distribution of fold‐change values was analysed with the Kolmogorov–Smirnov test. Pearson’s correlation coefficient was used to assess a possible linear association between two continuous quantitative variables. To determine the relationship between gene expression and RIN values according to pathologic variables, we used the analysis of covariance (ancova) in the 16 probes. Statistical analysis of the expression data between groups was made using one‐way analysis of variance (anova) followed by Tukey posttest, or Kruskal–Wallis test followed by Dunn’s post hoc test when required using the SPSS software (IBM Corp. Released 2013. IBM SPSS Statistics for Windows, Version 21.0; Armonk, NY: IBM Corp). Outliers were detected using the GraphPad software QuickCalcs (*P* < 0.05). All data were expressed as mean values ± SEM. Differences between MA and AD cases were considered statistically significant at *P < 0.05, **P < 0.01, ***P < 0.001 *vs*. MA; ^#^ *P* < 0.05, ^##^ *P* < 0.01, ^###^ *P* < 0.001 *vs*. ADI–II/0‐A and ^$^ *P* < 0.05 *vs*. ADIII–IV/0–C and ADV–VI/B–C.

### Immunohistochemistry

Formalin‐fixed, paraffin‐embedded, de‐waxed sections 4‐µm thick of the frontal white matter of cases listed in Table 1 were processed for specific immunohistochemistry. The sections were boiled in citrate buffer (20 min) to retrieve protein antigenicity. Endogenous peroxidases were blocked by incubation in 10% methanol, 1% H_2_O_2_ solution (15 min) followed by 3% normal horse serum solution. Then the sections were incubated at 4°C overnight with one of the primary rabbit polyclonal antibodies: NG2 (used at a dilution of 1:200; Sigma‐Aldrich, Merck, Darmstadt, GE), Olig2 (used at a dilution of 1:500, Sigma‐Aldrich, Merck); HDAC2 (diluted 1:100, Abcam, Cambridge, UK); GLUT 1 (diluted 1:100, Abcam); or with one of the primary mouse monoclonal antibodies: PLP1 (used at a dilution of 1:100, Lifespan Biosci, Seattle, WA, USA), CNPase (used at a dilution of 1:100, Sigma‐Aldrich, Merck), MBP (diluted 1:1000, Abcam) and GFAP (diluted 1:1000, Diagnostic BioSyst, CA, USA). Following incubation with the primary antibody, the sections were incubated with EnVision + system peroxidase (Dako, Agilent Technologies, Santa Clara, CA, USA) for 30 min at room temperature. The peroxidase reaction was visualized with diaminobenzidine and H_2_O_2_. Control of the immunostaining included omission of the primary antibody; no signal was obtained following incubation with only the secondary antibody. Sections were slightly counterstained with haematoxylin.

Quantification of NG2‐, Olig2‐ and HDAC2‐immunoreactive cells in the WM was done by counting the number of positive cells in areas of the WM located about 1 cm below the subcortical U fibres of the frontal lobe at the level of the head of the caudate and putamen chosen at random. Microphotographs were obtained at magnifications of × 200 or × 400, covering areas of 0.126 mm^2^ and 0.038 mm^2^, respectively, in three nonconsecutive sections per case using a DP25 camera adapted to an Olympus BX50 light microscope. The number of positive cells was counted directly on the figures and expressed as the number of positive cells per area (0.038 mm^2^ for NG2‐ and HDAC2‐immunoreactive cells, and 0.126 mm^2^ for Olig2‐positive cells). The normality of distribution of fold‐change values was analysed with the Kolmogorov–Smirnov test. Results were analysed with one‐way anova and post hoc Tukey; differences were considered statistically significant at * MA *vs*. AD; #: ADI–II *vs*. ADIII–IV or ADV–VI; $: ADIII–IV *vs*. ADV–VI; significance levels were set at: *^,^ # *P* < 0.05, **, ## *P* < 0.01, and ***, ###, $$$ *P* < 0.001. No attempts were made to quantify the densitometry of GLUT1.

Densitometric quantification of PLP1 immunoreactivity was acquired in the same areas as those indicated for NG2‐, Olig2‐ and HDAC2‐immunoreactive cells following a similar procedure to that described for Klüver–Barrera‐stained sections. Photomicrographs were obtained at a magnification of × 200, covering an area of 0.126 mm^2^, using a DP25 camera adapted to an Olympus BX50 light microscope_._ The pictures, two areas per case in every case, were analysed using Photoshop software. The density of PLP1 was calculated as the intensity of brown colour normalized by the total area excluding white spaces of the nuclei and expressed as arbitrary units per area. The normality of distribution was analysed with the Kolmogorov–Smirnov test. Results were analysed with one‐way anova and post hoc Tukey. Differences between MA and the three stages of pure AD cases were considered statistically significant at: * MA *vs*. AD; #: ADI–II *vs*. ADIII–IV or ADV–VI; $: ADIII–IV *vs*. ADV–VI. Significance levels were set at: *^,^ # *P* < 0.05, **, ## *P* < 0.01 and ***, ###, $$$ *P* < 0.001.

## Results

### White matter alterations in the general series

**Figure 1:**
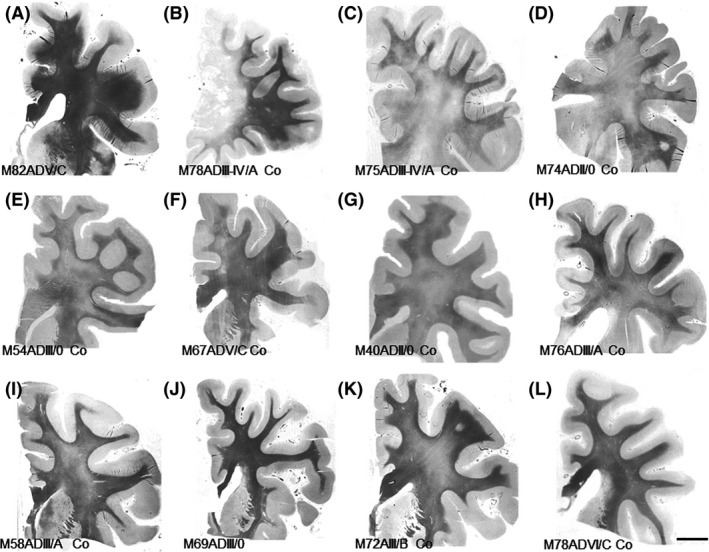
Representative formalin‐fixed, paraffin‐embedded, de‐waxed coronal sections of the frontal cortex at the level of the head of the caudate and putamen, stained with Klüver–Barrera. (**A**) AD stage V/C, the absence of co‐morbidities (M, 82y); (**B**) ADIII–IV/A presenting with a frontal infarct (M, 78y); (**C**) Patient categorized as mixed dementia suffering from HTA and extensive WM hyper‐lucencies, AD stage III–IV/A, LBD stage 3 (M, 75y); (**D**) Patient with chronic respiratory insufficiency and terminal hypoxia, AD pathology stage II/0 (M, 74y); (**E**) Patient with chronic respiratory failure, abnormal behaviour of nondetermined origin and AD‐ pathology stage III/0 (M, 54y); (**F**) Patient with cognitive impairment, focal WM hyper‐lucencies, lacunar infarcts, HTA and AD stage V/C (M, 67y); (**G**) Patient with cognitive impairment, HTA, WM hyper‐lucencies and AD pathology stage II/0 (M, 40y); (**H**) Patient with severe cognitive impairment, type II diabetes, hyperlipidaemia, obesity, HTA, renal failure, argyrophilic grain disease stage II and AD pathology III/A (M, 76y); (**I**) Patient with hepatic encephalopathy and AD pathology stage III/A (M, 58y); (**J**) Patient with no neurological symptoms, and the absence of clinical and pathological co‐morbidities, categorized as AD stage III/A (M, 69y); (**K**) Patient with mild cognitive impairment, WM hyper‐lucencies, HTA and AD pathology stage III/B (M, 72y); (**L**) Patient with long‐lasting dementia, the absence of risk factors of cerebral circulatory disturbance, and affected by ADVI/C, argyrophilic grain disease stage II and TDP‐43 proteinopathy (M, 76). The figure makes it evident that there is variable involvement of the WM in association with distinct cerebral and systemic disorders concomitant with AD pathology. M: man. Compare the variability of WM involvement in cases **B**, **C**, **E**, **H**, **I** and **K** categorized as AD with co‐morbidities (AD‐Co) with **J** categorized as AD without co‐morbidities, all of them stage ADIII–IV of Braak and Braak. Cases **D** and **G**, classified as AD‐Co stage II, also show decreased staining of the centrum semi‐ovale when compared with stage III of AD without co‐morbidities (**J**). Differences are also observed between AD without co‐morbidities stage V (**A**) in comparison with AD‐Co stages V and VI (**F** and **L**). Note that all cases in the figure correspond to males to avoid gender bias).

Representative examples of the diversity of WM changes in cases with AD pathology without and with co‐morbidities (AD‐Co) are shown in Figure 1. This illustrates the variability in WM alterations which may concur in cases with AD pathology.

**Figure 2:**
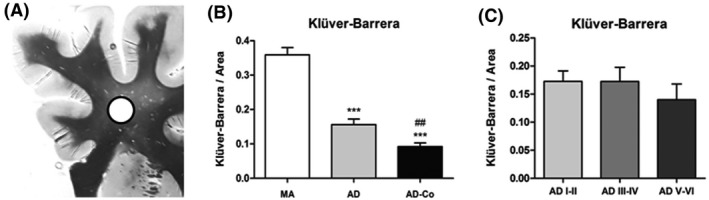
Densitometric values of myelin sheet phospholipids as revealed in Klüver–Barrera‐stained sections of the centrum semi‐ovale at the level of the head of the caudate and putamen in MA, AD with co‐morbidities (AD‐Co), and cases of AD without co‐morbidities (AD). Values are expressed as arbitrary units per area. (**A**) The area of densitometric studies is indicated by the circle; Klüver–Barrera staining in an MA case. Note that the area is separated from the periventricular white matter and the subcortical U‐fibres. (**B**) Significant decrease in AD without co‐morbidities (*n* = 33) and AD‐Co (*n* = 173) is seen when compared with MA (*n* = 20). The intensity of myelin staining is significantly lower in AD‐Co when compared with AD cases. (**C**) No significant differences, but a tendency to reduced myelin intensity is seen in AD without co‐morbidities at stages V–VI/B–C (ADV–VI) when compared with AD at stages I–II/0‐A (ADI–II) and AD at stages III–IV/0‐C (ADIII–IV). ADI–II/0‐A, *n* = 9; ADIII–IV/0–C, *n* = 8; ADV–VI/B–C, *n* = 16. One‐way anova and post hoc Tukey, ***P < 0.001 AD and AD‐Co compared with MA; ^##^ *P* < 0.001: AD‐Co compared with AD without co‐morbidities.

Densitometric studies of the central myelin were carried out in the AD series and in MA (Figure 2**A**). The density of myelin was significantly reduced in AD without co‐morbidities and in AD‐Co when compared with MA (*P* = 0.000 and *P* = 0.000 respectively). Myelin density was significantly lower in AD‐Co when compared with AD cases without co‐morbidities (*P* = 0.003) (Figure 2**B**).

Regarding AD cases without co‐morbidities, no significant differences, but rather a tendency to reduced myelin density was seen in AD stages V–VI when compared with AD stages I–II and AD stages III–IV (Figure 2**C**).

### RNA expression of genes related to oligodendroglia and myelin in WM of AD without co‐morbidities

**Figure 3:**
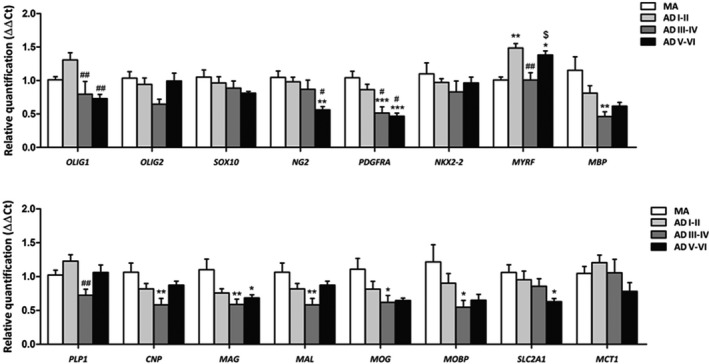
mRNA expression of selected oligodendrocyte‐ and myelin‐related genes in the frontal white matter of MA, AD without co‐morbidities stages I–II/0‐A (ADI–II), III–IV/0‐C (III–VI) and V–VI (**B**, **C**). Abbreviations may be seen in Table 2. One‐way analysis of variance (anova) followed by Tukey posttest or Kruskal–Wallis test followed by Dunn’s post hoc test when required using the SPSS software; *P < 0.05, **P < 0.01, ***P < 0.001 *vs*. MA; ^#^ *P* < 0.05, ^##^ *P* < 0.01 *vs*. ADI–II; ^$^ *P* < 0.05 *vs*. ADIII–IV and ADV–VI (see Methods for statistical studies).

Results of RT‐qPCR are summarized in Figure 3. The expression of several genes linked to the oligodendrocyte lineage was reduced at stages III–IV/0–C when compared with MA and with AD stages I–II/0‐A including *OLIG1* (*P* = 0.007, when compared with ADI–II/A–C) and *PDGFRA* (*P* = 0.000 when compared with MA, and *P* = 0.029 when compared with ADI–II/0‐A). Levels of *PDGFRA* were decreased at stages V–VI/B–C when compared with MA (*P* = 0.000); *PDGFRA* and *OLIG1* levels were also significantly decreased at stages V–VI/B–C when compared with levels at stages ADI–II/0‐A (*P* = 0.015 and *P* = 0.002 respectively). Curiously, *NG2* mRNA expression was reduced only at stages ADV–VI/B–C when compared with levels of MA and AD stages I–II/0‐C (*P* = 0.006 and *P* = 0.027 respectively). However, other markers of oligodendrocyte differentiation such as *SOX10* and *NKX‐2*, as well as *OLIG2*, were not significantly altered, although *OLIG2* showed a trend to decrease at middle stages of AD.

Regarding genes involved in myelin synthesis, *MYRF* mRNA expression, the product of which triggers myelination, was significantly increased in ADI–II/0‐A and ADV–VI/B–C when compared with MA (*P* = 0.002 and *P* = 0.033 respectively). *MYRF* expression was transiently reduced to normal levels at stages III–IV/0–C when compared with ADI–II/0‐A and ADV–VI/B–C (*P* = 0.005 and *P* = 0.049 respectively). In contrast, the majority of genes which encode proteins of the myelin sheet showed reduced expression levels at AD stages III–IV/0‐C when compared with MA or with AD stages I–II/0‐C: *MBP* (*P* = 0.009 when compared with MA), *PLP1* (*P* = 0.003 when compared with AD stages I–II/0‐A), and *CNP*, *MAG*, *MAL*, *MOG* and *MOBP* when compared with MA (*P* = 0.006, *P* = 0.005, *P* = 0.006, *P* = 0.035 and *P* = 0.041 respectively). Curiously, the mRNA expression levels of several myelin‐related genes were within normal values at AD stages V–VI/B–C, including *MBP*, *PLP1*, *CNP*, *MAL*, *MOG* and *MOBP*. Yet levels of *MAG* were decreased in ADV–VI/B–C when compared with MA (*P* = 0.032) (Figure 3).

The expression of the gene coding for glucose transporter (*SLC2A1*) was reduced in ADV–VI/B–C when compared with MA (*P* = 0.039). In contrast, the expression levels of *MCT1*, the gene coding for solute carrier family 16 (monocarboxylic acid transporter, member 1), were not significantly altered, but did show a trend to reduction at advanced stages of AD (Figure 3).

### Immunohistochemistry

**Figure 4:**
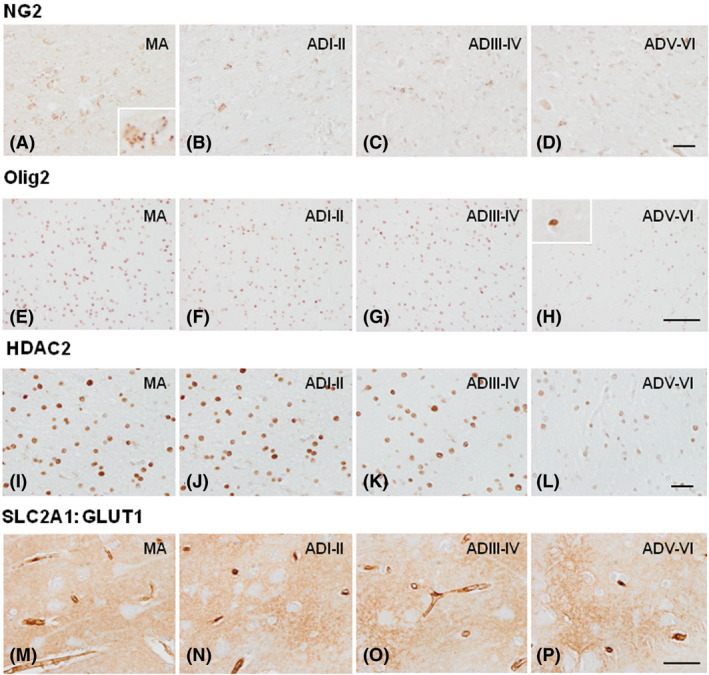
Immunohistochemistry to cellular markers NG2, Olig2, HDAC2 and SLC2A1:GLUT1 in MA individuals (**A**, **E**, **I**, **M**), and in cases with AD without co‐morbidities at stages ADI–II/0‐A (ADI–II) (**B**, **F**, **J**, **N**), ADIII–IV/0–C (ADIII–IV) (**C**, **G**, **K**, **O**) and ADV–VI/B–C (ADV–VI) (**D**, **H**, **L**, **P**). Decreased numbers of NG2‐, Olig2‐ and HDAC2‐immunoreactive cells are observed at middle, and particularly, advanced stages of AD. Large hyperchromatic Olig2‐positive cells are also observed in ADV–VI. GLUT1 immunoreactivity is manifested as a fine uniform meshwork in the neuropil which is progressively disrupted into patches of variable immunoreactivity with disease progression. Paraffin sections, slightly counterstained with haematoxylin; NG2, HDAC2, bar = 50 µm; Olig2 and SLC2A1, bar = 45 µm. Insert in MA NG2 is at greater magnification to show small positive granules characteristic of NG2 immunoreactivity. Insert in Olig2 ADV–VI shows a representative large hyperchromatic Olig2‐immunoreactive cell; these cells are commonly present in ADV–VI.

NG2 (which identifies oligodendroglial precursor cells) immunoreactivity in the WM was detected as small granules in the cytoplasm of a subpopulation of glial cells, whereas Olig2 (which is expressed in oligodendrocytes) immunoreactivity decorated the nucleus of oligodendrocytes. The number of NG2‐positive cells decreased with disease progression, but significant differences were detected only between ADI–II/0‐A when compared with ADIII–IV/0‐C (*P* = 0.001), and between ADI–II/0‐A compared with ADV–VI/B–C (*P* = 0.000). Olig2‐positive cells also decreased with disease progression: MA *vs*. ADIII–IV/0–C (*P* = 0.011), MA *vs*. ADV–VI/B–C (*P* = 0.000), ADI–II/0‐A *vs*. ADIII–IV/0–C (*P* = 0.016), ADI–II/0‐A *vs*. ADV–VI/B–C (*P* = 0.000) and ADIII–IV/0–C *vs*. ADV–VI/B–C (*P* = 0.000). Moreover, Olig2‐immunoreactive cells with large, dense and often elongated nuclei were found in ADV–VI/B–C (Figure 4, Figure 6).

HDAC2 immunoreactivity was found in the nucleus of glial cells. The number of HDAC2‐positive cells and the intensity of the staining in the remaining cells significantly decreased with disease progression: MA *vs*. ADIII–IV/0–C (*P* = 0.007), MA *vs*. ADV–VI/B–C (*P* = 0.000), ADI–II/0‐A *vs*. ADIII–IV/0‐C (*P* = 0.035); ADI–II/0‐A *vs*. ADV–VI/B–C (*P* = 0.000), and ADIII–IV/0‐C *vs*. ADV–VI/B–C (*P* = 0.000) (Figures 4 and 6).

GLUT1 (glucose transporter member 1) immunoreactivity, which decorated the neuropil and the wall of the small blood vessels, decreased in AD with disease progression. However, no attempt was made to quantify the density of the immunostaining due to individual variations (Figure 4).

**Figure 5:**
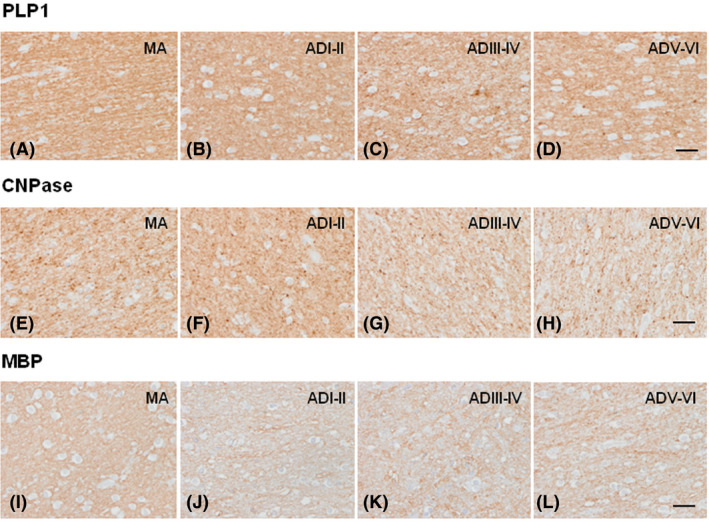
Immunohistochemistry to myelin markers PLP1, CNPase and MBP in the centrum semi‐ovale of the frontal lobe in MA (**A**, **E**, **I**), and in cases with AD without co‐morbidities at stages ADI–II/0‐A (ADI–II) (**B**, **F**, **J**), ADIII–IV/0‐C (ADIII–IV) (**C**, **G**, **K**) and ADV–VI/B–C (ADV–VI) (**D**, **H**, **L**). Representative images show reduced immunoreactivity with disease progression, and small PLP1‐ and CNPase‐immunoreactive dots in ADV–VI. Paraffin sections, slightly counterstained with haematoxylin; bar = 50 µm.

**Figure 6:**
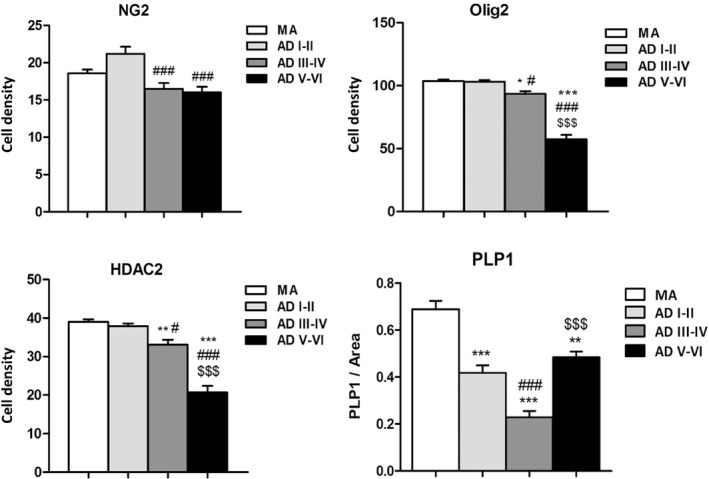
Quantitative study of NG2‐, Olig2‐ and HDAC2‐immunoreactive cells in the frontal WM per area of AD cases without co‐morbidities (0.038 mm^2^ for NG2‐ and HDAC2‐immunoreactive cells, and 0.126 mm^2^ for Olig2‐positive cells; see Methods). The number of positive cells decreases with increasing stages of AD pathology. MA, *n* = 10; ADI–II/0‐A, *n* = 9; ADIII–IV/0‐C, *n* = 8; ADV–VI/B–C, *n* = 16. One‐way anova and post hoc Tukey; *: MA *vs*. ADIII–IV/0‐C (ADIII–IV) or ADV‐VI/B–C (ADV–VI); #: ADI–II/0‐A (ADI–II) *vs*. ADIII–IV or ADV–VI; $: ADIII–IV *vs*. ADV–VI; significance level set at *^,^^#^ *P* < 0.05, ** *P* < 0.01 and ***, ^###^, ^$$$^ *P* < 0.001.

Regarding myelin proteins, the intensity of PLP1, CNPase and MBP immunoreactivity decreased with disease progression (Figure 5). Densitometric studies were carried out only for PLP1. Significant reduction was observed in ADI–II/0‐A, ADIII–IV/0–C and ADV–VI/B–C when compared with MA (*P* = 0.000, *P* = 0.000 and *P* = 0.002 respectively). Moreover, significant differences were also seen between ADI–II/0‐ *vs*. ADIII–IV/0‐C (*P* = 0.000) and ADIII–IV/0‐C *vs*. ADV–VI/B–C (*P* = 0.000) (Figure 6).

In contrast, GFAP immunoreactivity showed an increase in the number and intensity in individual astrocytes in the WM in parallel sections (data not shown), in agreement with previous data from several authors, as reviewed elsewhere [78].

## Discussion

This study was designed to learn about WM abnormalities in cases with AD pathology without co‐morbidities at different stages of disease progression. For this purpose, our first approach was to consider all cases in which one of the *post mortem* neuropathological diagnoses was AD pathology in a continuous series of necropsy cases in a general hospital. Following this procedure, 206 of the 470 cases with adequate clinical information and histological quality to carry out densitometric studies of the WM of the frontal lobe were selected. Interestingly, 89% of cases aged 65 and older had AD pathology, a slightly higher figure than the percentage already reported for this age group [79, 80]. Review of the clinical history and neuropathological study revealed that 84% had concomitant cerebrovascular pathology; clinical morbidities such as HTA, type II diabetes, hyperlipaemia, renal or liver failure and chronic respiratory failure; cerebrovascular pathology; age‐related neurodegenerative diseases, such as other tauopathies, Lewy body pathology and TDP‐43 proteinopathy; and long agonic state, or vegetative state, which were putative causes of WM alterations. After further selection, 33 were categorized as AD cases without co‐morbidities.

It can be argued that not all AD cases had cognitive impairment and dementia, and therefore, they cannot be classified as AD. Moreover, six cases of AD stages I–III without co‐morbidities did not have β‐amyloid deposits, and they might be classified as Primary age‐related tauopathy (PART) [81]. However, PART has also been considered as part of AD [82]. We have followed here this consideration because AD and PART share the same NFT pathology at early stages of the disease.

Densitometric analysis performed on Klüver–Barrera‐processed sections of the central WM of the frontal lobe at the level of the head of caudate and putamen revealed significant differences between MA individuals and cases with AD. It is important to stress that the MA group was composed of individuals with an age of about 25 years younger than that of the AD groups. Therefore, MA must be not considered an age‐matched control group, but rather a representation of the myelin in normal MA individuals. A significant reduction in phospholipid myelin in elderly, as revealed with Klüver–Barrera staining, is consistent with the idea that myelin in the cerebral WM decreases with age, as already noted in the Introduction. Since the majority of individuals aged 65 and older had AD pathology, it is difficult to ascertain whether changes in the AD groups were linked to ageing or associated with AD pathology.

More practical is the observation that myelin decay is greater in AD cases with co‐morbidities (AD‐Co) than in AD cases with no co‐morbidities. The age of the individuals has been assessed in every case, and the mean values ± SEM have been obtained for every stage both in the total series and in pure AD. Values are similar in both groups, and then comparisons are not biased by differences in the age, but in the presence or absence of co‐morbidities. Since the majority of cases with AD in our series had co‐morbidities, it may be inferred that part of the WM lesions commonly reported in the elderly and in AD during life cannot be ascribed solely to age and AD, but rather to common concomitant risk factors and pathologies affecting the ageing brain.

### Biochemical alterations in WM in AD without co‐morbidities

Previous studies have shown alterations in the lipid composition of the WM in AD. Galactosylceramide (GalCer) and sulphatides, synthesized by oligodendroglia in the CNS, are major components of myelin. Reduced GalCer and sulphatide levels, increased cholesterol and increased fatty acid contents occur in cortical grey and WM in AD [83, 84, 85, 86, 87, 88]. Levels of GalCer and sulphatide slightly decreased in the frontal and temporal cortex, and in WM matter at stages III–IV, and more markedly at stages V–VI in AD [89]. Curiously, the activity of ceramide synthase 2, which catalyses the synthesis of very long chain ceramides, decreased in brain temporal cortex at stages I–II and frontal cortex at stages III–IV preceding neurofibrillary tangle formation, suggesting that alterations of ceramide synthesis occur earlier than previously suspected in the spread of AD [89]. Our observations on Klüver–Barrera‐stained sections are in line with biochemical studies showing decreased levels of phospholipid components of myelin in AD when compared with MA individuals. Differences are not as clear among stages in AD cases without co‐morbidities, although a trend to reduction is observed at advanced stages.

Regarding myelin proteins, progressive reduction in the levels of myelin basic protein (MBP), myelin proteolipid protein (PLP) and 2′,3′‐cyclic nucleotide 3′‐phosphodiesterase (CNP) has been reported in the WM of the parietal and occipital lobes in AD correlating with Braak stages V–VI [90]. Reduced levels of CNP have also been recorded in the WM of the frontal lobes in advanced AD [89, 90].

Our observations are restricted to cases with AD pathology without co‐morbidities. mRNA expression levels of *MBP*, *PLP1*, *CNP*, *MAG*, *MAL*, *MOG* and *MOBP* were preserved in the frontal white matter at stages I–II/0‐A when compared with MA, but they were transiently decreased at stages III–IV/0‐C, and increased thereafter to reach nearly MA levels at stages V–VI/B–C. Regarding protein expression, densitometric studies of PLP1 in the same region revealed a significant decrease in PLP1 immunoreactivity at early stages of AD, which became more marked at middle stages, followed by a slight increase without reaching MA values at advanced stages of AD.

It is worth to stress that differences between AD stages are not related to the age or gender of the individuals, but rather to the stage of the disease. Differences between myelin lipids, as revealed with Klüver–Barrera staining, and myelin proteins as shown by RT‐qPCR and immunohistochemistry, in the same cases may be related to differing preciseness of the methods employed. However, the relative recovery of myelin proteins, but not of phospholipids, at advanced stages of the disease deserves further study, including analysis of a possible structural imbalance between lipid and protein components of the myelin sheet in the elderly and in AD. Studies in the aged human *post mortem* brain have shown decreased internodal distance, reduced axon thickness and greater vulnerability of thin myelinated fibres compared with large myelinated fibres [7]. Little is known about this aspect in AD.

### β‐amyloid and tau

It has been suggested that cortical atrophy with neuron loss is not the main cause of WM damage in AD, as revealed by pioneering neuropathological studies [29], and later supported by combined MRI and *post mortem* examination [9, 91]. However, this hypothesis has been brought into question by other studies. The impact of β‐amyloid on oligodendrocytes is equivocal. On the one hand, β‐amyloid is toxic to oligodendrocytes as identified in *in vitro* models, transgenic mice and familial AD [92, 93, 94, 95, 96]. However, Aβ oligomers also promote oligodendrocyte differentiation and maturation in isolated oligodendrocytes and in organotypic cerebellar slices [97]. In fact, deterioration of the WM parallels, but does not correlate with either the total amount or the regional localization of β‐amyloid plaques [98]. However, soluble β‐amyloid is abundant in the WM in the absence of plaques in AD [99]. Therefore, the possibility of oligodendrocyte damage by soluble β‐amyloid in AD cannot be ignored.

WM damage has been correlated with tau pathology in the cerebral cortex [100, 101]. Therefore, myelin breakdown in AD has been posited as being linked to axonopathy and transport deficits [100, 101, 102]. In favour of WM axonopathy resulting from tau pathology is the presence of WM breakdown in transgenic mice bearing the P301L mutation in the *mapt* gene [13]. Yet, WM disruption does not correlate exactly with the localization and distribution of NFTs in AD [29]. The present findings further support the concept of early alteration of the WM in AD, as transcription of oligodendrocyte and myelin genes in the frontal WM is altered at stages III–IV/0–C at which point no NFTs or neuron loss are found in this region.

### Oligodendrocytes and NG2‐glia

Previous studies have shown reduced size of the nuclei of oligodendrocytes [14], and decreased numbers of Olig2‐ and NG2‐glia‐immunoreactive cells [105, 106]. Moreover, several oligodendroglial nuclei in the WM show oxidative damage (8‐OHdG immunoreactivity), whereas other oligodendrocytes exhibit increased expression of p53 as a marker of stress, and a senescent phenotype (SA‐β‐gal immunoreactive) [107, 108].

Reduction in the expression of *NG2* and *PDGFRA* mRNA (stages V–VI/A–C, and stages III–IV/0–C and V–VI/A–C respectively), together with reduced numbers of NG2‐ and Olig2‐immunoreactive cells in the WM, points to progressive decline of the oligodendrocyte lineage in the frontal WM with disease progression, which is in line with the demonstration of early alterations of the oligodendrocyte lineage linked to AD pathology [105, 106].

Reduction in NG2‐glia is important as it probably compromises the regenerative ability of the WM to restore the number of oligodendrocytes and myelin homeostasis. Reduced numbers of oligodendrocytes and altered control of glucose and lactate metabolites necessary for trophic support for axons may produce, in turn, altered axonal function, and axonal degeneration. This scenario takes place at a limited rate in the ageing human brain [19], but its potentially damaging effect is overwhelmed in AD [110].

Histone deacetylases (HDACs) remove specific acetyl groups on a histone enabling it to interact with DNA thereby modulating gene transcription. Increased HDAC2 facilitates oligodendrocyte differentiation [73, 74]. Thus, HDAC2 reduction likely compromises oligodendrogenesis.

An intriguing feature in this scenario is the increased expression of *MYRF* mRNA at early and late stages of AD. Regarding the number of oligodendrocytes, reduced expression of *MYRF* mRNA could be expected, whereas with MYRF being a factor regulating the expression of several myelin genes, the expression of such genes would be expected to increase in AD. We have no explanation for the altered MYRF regulation and response in AD.

### Transgenic mice expressing β‐amyloid and tau as animal models to uncover WM abnormalities in AD

WM alterations have also been examined in several types of transgenic mice carrying the APP/PS1 (K670N/M671L Swedish and PS1dE9) mutation, PS1 mutation, and 5xFAD mutation (Swedish mutation, 1716V Florida mutation, V717I London mutation, and PS1 with M146L and L286V mutation), as well as 3xTg‐AD mice (APP Swedish mutation, a presenilin knock‐in mutation and P301L tau) [111, 112, 113, 114, 115, 116, 117].

In all these models, WM alterations characterized by myelin loss and decreased numbers of oligodendrocytes occur at early stages before the appearance of β‐amyloid plaques, and they increase for a limited period of disease progression. Oligodendroglial cell death and myelin loss occur at early stages in 3xTg‐AD mice [112, 113], and region‐specific alterations appear before β‐amyloid and tau pathology [112]. However, murine models of β‐amyloid deposition show reparative responses at later stages of the disease [105, 114, 115, 116]. No similar increase in Olig2 and NG2‐immunoreactive cells occurs in AD, as shown in the present work and in previous studies [105, 106, 109, 110]. However, a certain normalization of the mRNA expression of several myelin‐related genes here observed in AD without co‐morbidities has its counterpart in transgenic models. As a working hypothesis, it may be suggested that repair of oligodendrocyte lineage is activated in AD and transgenic models, but regeneration is minimized in AD when compared with transgenic murine models.

### Vascular alterations in ageing and AD

Vascular and circulatory alterations including atherosclerosis, small blood vessel disease, tortuous and coiled arterioles, reduced vascular density and cerebral complications such as micro‐infarcts, hypoperfusion and micro‐bleeds are common in aged human brains [4, 118, 119, 120, 121, 122, 123].

In addition to vascular alterations linked to age, which may occur in any patient, hypoperfusion linked to β‐amyloid deposition, alteration of the blood vessel walls, increased RAGE, altered microglia and astrocytes with senescent forms are constant in AD. β‐amyloid angiopathy is found in the vast majority of cases [124, 125] and it shows an early predilection for cortical blood vessels. β‐amyloid deposition is accompanied by decreased expression of efflux receptor for β‐amyloid and increased influx receptor RAGE in AD [126, 127]. Other alterations include atrophy, oedema and increased numbers of pinocytic vesicles in endothelial cells; thickening and focal disruption of the basal membrane; increase in heparan sulphate, proteoglycans, collagen IV and laminin in the basal membrane, with atrophy of smooth muscle fibres and augmented aquaporin expression in perivascular astrocytes [128, 129, 130, 131, 132]. These changes, in conjunction with mounting evidence of altered blood–brain barrier in AD [133, 134, 135, 136], lend support to the old proposal of reduced perfusion of the brain as a pathogenic factor in AD [137, 138, 139].

Our study in AD cases without co‐morbidities excludes major vascular pathology and systemic circulatory factors which could have an impact on the integrity of oligodendrocytes and myelin. However, we cannot rule out a role for primary alterations in blood vessels linked to AD since specific vascular pathology is one of the manifestations of AD.

## Conclusions

WM alterations in AD have been considered the result of cerebrovascular dysfunction [138, 139], axonopathy linked to retrograde neuronal tau deposition [100, 101, 102], or homeostatic responses to age‐related myelin breakdown [140]. These factors are not mutually exclusive, but rather reinforce each other with age and disease progression. This study has tried to minimize co‐morbidities in a general population with AD pathology dying in a general hospital. It may be argued that: i. the population dying in a general hospital is not representative of the total population whose health status is better than that represented by in‐patients, and that co‐morbidities are less frequent and serious in the general population when compared with individuals dying in the hospital; and ii. vascular ageing, and of course vascular changes linked to AD, cannot be ruled out in the pathogenesis of WM disorders in the present series of AD cases without co‐morbidities. These are undoubtedly reasonable objections.

Yet, several studies have stressed the role of oligodendrocytes as important players in the pathogenesis of distinct neurodegenerative diseases including AD [141, 142, 143, 144]. In this line, the present observations point to early alterations of oligodendrocytes and transcription of genes linked to myelin proteins in WM in cases with AD pathology without co‐morbidities before the appearance of NFTs in these regions, and before the appearance of clinical symptoms of cognitive impairment. This suggests that oligodendrocytes are along with neurons, targets of AD, and that oligodendrocytopathy is therefore part of AD.

## Author contributions

IF designed the study, revised the neuropathology of cases, selected the cases without co‐morbidities, interpreted the results and wrote the manuscript; PAB carried out the RT‐qPCR studies and immunohistochemistry. Both authors revised and approved the final version of the manuscript.

## Disclosure

The authors declare that they do not have conflict of interest.

## Funding information

The project leading to these results received funding from ‘la Caixa’ Foundation under the agreement LCF/PR/HR19/52160007. The study was also supported by the Ministry of Economy and Competiveness, Institute of Health Carlos III (cofunded by European Regional Development Fund, ERDF, a way to build Europe): FIS PI17/000809 and IFI15/00035 fellowship to PA‐B. We thank CERCA Programme/Generalitat de Catalunya for institutional support.

## Data Availability

Data sharing is not applicable to this article as no new data were created or analysed in this study.

## References

[1] Hachinski VC , Potter P , Merskey H . Leuko‐araiosis. Arch Neurol 1987; 44: 21–3 3800716 10.1001/archneur.1987.00520130013009

[2] Scheltens P , Barkhof F , Leys D , Wolters EC , Ravid R , Kamphorst W . Histopathologic correlates of white matter changes on MRI in Alzheimer's disease and normal aging. Neurology 1995; 45: 883–8 7746401 10.1212/wnl.45.5.883

[3] Barber R , Scheltens P , Gholkar A , Ballard C , McKeith I , Ince P , et al. White matter lesions on magnetic resonance imaging in dementia with Lewy bodies, Alzheimer's disease, vascular dementia, and normal aging. J Neurol Neurosurg Psychiat 1999; 67: 66–72 10369824 10.1136/jnnp.67.1.66 PMC1736409

[4] Holland CM , Smith EE , Csapo I , Gurol ME , Brylka DA , Killiany RJ , et al. Spatial distribution of white‐matter hyperintensities in Alzheimer disease, cerebral amyloid angiopathy, and healthy aging. Stroke 2008; 39: 1127–33 18292383 10.1161/STROKEAHA.107.497438 PMC2754400

[5] Salat DH , Greve DN , Pacheco JL , Quinn BT , Helmer KG , Buckner RL , et al. Regional white matter volume differences in non‐demented aging and Alzheimer’s disease. NeuroImage 2009; 44: 1247–58 19027860 10.1016/j.neuroimage.2008.10.030 PMC2810540

[6] Liu H , Yang Y , Xi Y , Zhu W , Leak RK , Wei Z , et al. Aging of cerebral white matter. Ageing Res Rev 2017; 34: 64–76 27865980 10.1016/j.arr.2016.11.006 PMC5250573

[7] Marner L , Nyengaard JR , Tang Y , Pakkenberg B . Marked loss of myelinated nerve fibers in the human brain with age. J Comp Neurol 2003; 462: 144–52 12794739 10.1002/cne.10714

[8] Gao J , Cheung RT , Lee TM , Chu LW , Chan YS , Mak HK , et al. Possible retrogenesis observed with fiber tracking: an anteroposterior pattern of white matter disintegrity in normal aging and Alzheimer’s disease. J Alzh Dis 2011; 26: 47–58 10.3233/JAD-2011-101788 21558648

[9] Erten‐Lyons D , Woltjer R , Kaye J , Mattek N , Dodge HH , Green S , et al. Neuropathologic basis of white matter hyperintensity accumulation with advanced age. Neurology 2013; 81: 977–83 23935177 10.1212/WNL.0b013e3182a43e45 PMC3888199

[10] Wharton SB , Simpson JE , Brayne C , Ince PG . Age‐associated white matter lesions: the MRC Cognitive Function and Ageing Study. Brain Pathol 2015; 25: 35–43 25521175 10.1111/bpa.12219 PMC8029351

[11] Kavroulakis E , Simos PG , Kalaitzakis GA , Maris TG , Karageorgou D , Zaganas I , et al. Myelin content changes in probable Alzheimer’s disease and mild cognitive impairment: associations with age and severity of neuropsychiatric impairment. J Magn Reson Imaging 2018; 47: 1359–72 28861929 10.1002/jmri.25849

[12] Bartzokis G , Cummings JL , Sultzer D , Henderson VW , Nuechterlein KH , Mintz J . White matter structural integrity in healthy aging adults and patients with Alzheimer disease: a magnetic resonance imaging study. Arch Neurol 2003; 60: 393–8 12633151 10.1001/archneur.60.3.393

[13] Bartzokis G , Sultzer D , Lu PH , Nuechterlein KH , Mintz J , Cummings JL . Heterogeneous age‐related breakdown of white matter structural integrity: implications for cortical “disconnection” in aging and Alzheimer’s disease. Neurobiol Aging 2004; 25: 843–51 15212838 10.1016/j.neurobiolaging.2003.09.005

[14] Bennett IJ , Madden DJ . Disconnected aging: cerebral white matter integrity and age‐related differences in cognition. Neuroscience 2014; 276: 187–205 24280637 10.1016/j.neuroscience.2013.11.026 PMC4032380

[15] Sugiyama I , Tanaka K , Akita M , Yoshida K , Kawase T , Asou H . Ultrastructural analysis of the paranodal junction of myelinated fibers in 31‐month‐old‐rats. J Neurosci Res 2002; 70: 309–17 12391590 10.1002/jnr.10386

[16] Kohama SG , Rosene DL , Sherman LS . Age‐related changes in human and non‐human primate white matter: from myelination disturbances to cognitive decline. Age 2012; 34: 1093–110 22203458 10.1007/s11357-011-9357-7 PMC3448998

[17] Diaz JF , Merskey H , Hachinski VC , Lee DH , Boniferro M , Wong CJ , et al. Improved recognition of leukoaraiosis and cognitive impairment in Alzheimer's disease. Arch Neurol 1991; 48: 1022–25 1929892 10.1001/archneur.1991.00530220038016

[18] Rose SE , Chen F , Chalk JB , Zelaya FO , Strugnell WE , Benson M , et al. Loss of connectivity in Alzheimer's disease: an evaluation of white matter tract integrity with colour coded MR diffusion tensor imaging. J Neurol Neurosurg Psychiatry 2000; 69: 528–30 10990518 10.1136/jnnp.69.4.528 PMC1737121

[19] Wang L , Goldstein FC , Levey AI , Lah JJ , Meltzer CC , Holder CA , et al. White matter hyperintensities and changes in white matter integrity in patients with Alzheimer’s disease. Neuroradiology 2011; 53: 373–81 21152911 10.1007/s00234-010-0806-2 PMC3332065

[20] Radanovic M , Pereira FR , Stella F , Aprahamian I , Ferreira LK , Forlenza OV , et al. White matter abnormalities associated with Alzheimer's disease and mild cognitive impairment: a critical review of MRI studies. Expert Rev Neurother 2013; 13: 483–93 23621306 10.1586/ern.13.45

[21] Brickman AM . Contemplating Alzheimer's disease and the contribution of white matter hyperintensities. Curr Neurol Neurosci Rep 2013; 13: 415 24190781 10.1007/s11910-013-0415-7 PMC3874404

[22] Joki H , Higashiyama Y , Nakae Y , Kugimoto C , Doi H , Kimura K , et al. White matter hyperintensities on MRI in dementia with Lewy bodies, Parkinson’s disease with dementia, and Alzheimer’s disease. J Neurol Sci 2018; 385: 99–104 29406924 10.1016/j.jns.2017.12.018

[23] Medina D , Urresta F , Gabrieli JD , Moseley M , Fleischman D , Bennett DA , et al. White matter changes in mild cognitive impairment and AD: a diffusion tensor imaging study. Neurobiol Aging 2006; 27: 663–72 16005548 10.1016/j.neurobiolaging.2005.03.026

[24] Selnes P , Fjell AM , Gjerstad L , Bjornerud A , Wallin A , Due‐Tonnessen P , et al. White matter imaging changes in subjective and mild cognitive impairment. Alzheimers Dement 2012; 8: S112–21 23021621 10.1016/j.jalz.2011.07.001

[25] Molinuevo JL , Ripolles P , Simo M , Llado A , Olives J , Balasa M , et al. White matter changes in preclinical Alzheimer's disease: a magnetic resonance imaging diffusion tensor imaging study on cognitively normal older people with positive amyloid beta protein 42 levels. Neurobiol Aging 2014; 35: 2671–2680 25002037 10.1016/j.neurobiolaging.2014.05.027

[26] Hoy AR , Ly M , Carlsson CM , Okonkwo OC , Zetterberg H , Blennow K , et al. Microstructural white matter alterations in preclinical Alzheimer's disease detected using free water elimination diffusion tensor imaging. PLoS One 2017; 12: e0173982 28291839 10.1371/journal.pone.0173982 PMC5349685

[27] Bouhrara M , Reiter DA , Bergeron CM , Zukley LM , Ferrucci L , Resnick SM , Spencer RG . Evidence of demyelination in mild cognitive impairment and dementia using a direct and specific magnetic resonance imaging measure of myelin content. Alzheimers Dement 2018; 14: 998–1004 29679574 10.1016/j.jalz.2018.03.007 PMC6097903

[28] Lee S , Viqar F , Zimmerman ME , Narkhede A , Tosto G , Benzinger TL , et al. White matter hyperintensities are a core feature of Alzheimer's disease: evidence from the dominantly inherited Alzheimer network. Ann Neurol 2016; 79: 929–39 27016429 10.1002/ana.24647 PMC4884146

[29] de la Monte SM . Quantitation of cerebral atrophy in preclinical and end‐stage Alzheimer’s disease. Ann Neurol 1989; 25: 450–59 2774485 10.1002/ana.410250506

[30] Sjobeck M , Haglund M , Englund E . Decreasing myelin density reflected increasing white matter pathology in Alzheimer’s disease – a neuropathological study. Int J Geriatr Psychiatry 2005; 20: 919–26 16163742 10.1002/gps.1384

[31] Gouw AA , Seewann A , Vrenken H , van der Flier WM , Rozemuller JM , Barkhof F , et al. Heterogeneity of white matter hyperintensities in Alzheimer's disease: post‐mortem quantitative MRI and neuropathology. Brain 2008; 131: 3286–98 18927145 10.1093/brain/awn265

[32] Ihara M , Polvikoski TM , Hall R , Slade JY , Perry RH , Oakley AE , et al. Quantification of myelin loss in frontal lobe white matter in vascular dementia, Alzheimer’s disease, and dementia with Lewy bodies. Acta Neuropathol 2010; 119: 579–89 20091409 10.1007/s00401-009-0635-8 PMC2849937

[33] Reisberg B , Franssen EH , Souren LE , Auer SR , Akram I , Kenowsky S . Evidence and mechanisms of retrogenesis in Alzheimer's and other dementias: management and treatment import. Am J Alzheimers Dis Other Demen 2002; 17: 202–12 12184509 10.1177/153331750201700411 PMC10833976

[34] Benitez A , Fieremans E , Jensen JH , Falangola MF , Tabesh A , Ferris SH , et al. White matter tract integrity metrics reflect the vulnerability of late‐myelinating tracts in Alzheimer's disease. Neuroimage Clin 2014; 4: 64–71 24319654 10.1016/j.nicl.2013.11.001 PMC3853114

[35] Jellinger KA . The enigma of vascular cognitive disorder and vascular dementia. Acta Neuropathol 2007; 113: 349–88 17285295 10.1007/s00401-006-0185-2

[36] Ferrer I . Cognitive impairment of vascular origin: neuropathology of cognitive impairment. J Neurol Sci 2010; 299: 139–49 20846674 10.1016/j.jns.2010.08.039

[37] Thal DR , Grinberg TL , Attems J . Vascular dementia: different forms of vessel disorders contribute to the development of dementia in the elderly brain. Exp Gerontol 2012; 47: 816–24 22705146 10.1016/j.exger.2012.05.023 PMC3470831

[38] Kalaria RN . Neuropathological diagnosis of vascular cognitive impairment and vascular dementia with implications for Alzheimer's disease. Acta Neuropathol 2016; 131: 659–85 27062261 10.1007/s00401-016-1571-z PMC4835512

[39] McAleese KE , Alafuzoff I , Charidimou A , De Reuck J , Grinberg LT , Hainsworth AH , et al. Post‐mortem assessment in vascular dementia: advances and aspirations. BMC Med 2016; 14: 129 27600683 10.1186/s12916-016-0676-5 PMC5011905

[40] Skrobot OA , Attems J , Esiri M , Hortobágyi T , Ironside JW , Kalaria RN , et al. Vascular cognitive impairment neuropathology guidelines (VCING): the contribution of cerebrovascular pathology to cognitive impairment. Brain 2016; 139: 2957–69 27591113 10.1093/brain/aww214

[41] Khan A , Kalaria RN , Corbett A , Ballard C . Update on vascular dementia. J Geriatr Psychiatry Neurol 2016; 29: 281–301 27502303 10.1177/0891988716654987

[42] Vinters HV , Zarow C , Borys E , Whitman JD , Tung S , Ellis WG , et al. Review: vascular dementia: clinicopathologic and genetic considerations. Neuropathol Appl Neurobiol 2018; 44: 247–66 29380913 10.1111/nan.12472

[43] Delay J , Brion S . Les Demences Tardives. Paris: Masson & Cie, 1962

[44] Tomlinson BE , Blessed G , Roth M . Observations on the brains of demented people. J Neurol Sci 1970; 11: 205–42 5505685 10.1016/0022-510x(70)90063-8

[45] Snowdon DA , Greiner LH , Mortimer JA , Riley KP , Greiner PA , Markesbery WR . Brain infarction and the clinical expression of Alzheimer's disease. The Nun Study. JAMA 1997; 277: 813–17 9052711

[46] Schneider JA , Wilson RS , Bienias JL , Evans DA , Bennett DA . Cerebral infarction and the likelihood of dementia from Alzheimer disease pathology. Neurology 2004; 62: 1148–55 15079015 10.1212/01.wnl.0000118211.78503.f5

[47] Chui HC , Zarow C , Mack WJ , Elis WG , Zheng L , Jagust WJ , et al. Vinters HV. Cognitive impact of subcortical vascular and Alzheimer's disease pathology. Ann Neurol 2006; 60: 677–87 17192928 10.1002/ana.21009 PMC1851933

[48] Jellinger KA . The enigma of mixed dementia. Alzheimers Dement 2007; 3: 40–53 19595916 10.1016/j.jalz.2006.09.002

[49] Ferrer I , Vidal N . Neuropathology of cerebrovascular diseases. Handb Clin Neurol 2017; 145: 79–114 28987197 10.1016/B978-0-12-802395-2.00007-9

[50] Magaki S , Yong WH , Khanlou N , Tung S , Vinters HV . Comorbidity in dementia: update of an ongoing autopsy study. J Am Geriatr Soc 2014; 62: 1722–8 25039832 10.1111/jgs.12977 PMC4172655

[51] White LR , Edland SD , Hemmy LS , Montine KS , Zarow C , Sonnen JA , et al. Neuropathologic comorbidity and cognitive impairment in the Nun and Honolulu‐Asia Aging Studies. Neurology 2016; 86: 1000–8 26888993 10.1212/WNL.0000000000002480 PMC4799714

[52] Haaksma ML , Vilela LR , Marengoni A , Calderón‐Larrañaga A , Leoutsakos JS , Olde Rikkert MGM , et al. Comorbidity and progression of late onset Alzheimer’s disease: a systematic review. PLoS One 2017; 12: e0177044 28472200 10.1371/journal.pone.0177044 PMC5417646

[53] Alafuzoff I , Kovacs GG . Comorbidities. Handb Clin Neurol 2017; 145: 573–7 28987195 10.1016/B978-0-12-802395-2.00036-5

[54] Karki R , Kodamullil AT , Hofmann‐Apitius M . Comorbidity analysis between Alzheimer’s disease and type 2 diabetes mellitus based on shared pathways and the role of T2DM drugs. J Alzheimers Dis 2017; 60: 721–31 28922161 10.3233/JAD-170440 PMC5611890

[55] Wang JH , Wu YJ , Tee BL , Lo RY . Medical comorbidity in Alzheimer's disease: a nested case‐control study. J Alzheimers Dis 2018; 63: 773–81 29660933 10.3233/JAD-170786

[56] Mendes A , Tezenas du Montcel S , Levy M , Bertrand A , Habert MO , Bertin H , et al. Multimorbidity is associated with preclinical Alzheimer's disease neuroimaging biomarkers. Dement Geriatr Cogn Disord 2018; 45: 272–81 29953971 10.1159/000489007

[57] Tripathi RB , Rivers LE , Young KM , Jamen F , Richardson WD . NG2 glia generate new oligodendrocytes but few astrocytes in a murine experimental autoimmune encephalomyelitis model of demyelinating disease. J Neurosci 2010; 30: 16383–90 21123584 10.1523/JNEUROSCI.3411-10.2010 PMC3063541

[58] Zawadzka M , Rivers LE , Fancy SP , Zhao C , Tripathi R , Jamen F , et al. CNS‐resident glial progenitor/stem cells produce Schwann cells as well as oligodendrocytes during repair of CNS demyelination. Cell Stem Cell 2010; 6: 578–90 20569695 10.1016/j.stem.2010.04.002 PMC3856868

[59] Tognata R , Miller RH . Contribution of the oligodendrocyte lineage to CNS repair and neurodegenerative pathologies. Neuropharmacology 2016; 110: 539–47 27108096 10.1016/j.neuropharm.2016.04.026 PMC5512544

[60] Liu Z , Hu X , Cai J , Liu B , Peng X , Wegner M , et al. Induction of oligodendrocyte differentiation by Olig2 and Sox10: evidence for reciprocal interactions and dosage‐dependent mechanisms. Developl biol 2007; 302: 683–93 10.1016/j.ydbio.2006.10.007 17098222

[61] Cahoy JD , Emery B , Kaushal A , Foo LC , Zamanian JL , Christopherson KS , et al. A transcriptome database for astrocytes, neurons, and oligodendrocytes: a new resource for understanding brain development and function. J Neurosci 2008; 28: 264–78 18171944 10.1523/JNEUROSCI.4178-07.2008 PMC6671143

[62] Emery B , Agalliu D , Cahoy JD , Watkins TA , Dugas JC , Mulinyawe SB , et al. Myelin gene regulatory factor is a critical transcriptional regulator required for CNS myelination. Cell 2009; 138: 172–185 19596243 10.1016/j.cell.2009.04.031 PMC2757090

[63] Jahn O , Tenzer S , Werner HB . Myelin proteomics: molecular anatomy of an insulating sheath. Mol Neurobiol 2009; 40: 55–72 19452287 10.1007/s12035-009-8071-2 PMC2758371

[64] Llorens F , Gil V , del Río JA . Emerging functions of myelin‐associated proteins during development, neuronal plasticity, and neurodegeneration. FASEB J 2011; 25: 463–75 21059749 10.1096/fj.10-162792

[65] Koenning M , Jackson S , Hay CM , Faux C , Kilpatrick TJ , Willingham M , Emery B . Myelin gene regulatory factor is required for maintenance of myelin and mature oligodendrocyte identity in the adult CNS. J Neurosci 2012; 32: 12528–42 22956843 10.1523/JNEUROSCI.1069-12.2012 PMC3752083

[66] Bujalka H , Koenning M , Jackson S , Perreau VM , Pope B , Hay CM , et al. MYRF is a membrane‐associated transcription factor that autoproteolytically cleaves to directly activate myelin genes. PLoS Biol 2013; 11: e1001625 23966833 10.1371/journal.pbio.1001625 PMC3742440

[67] Raasakka A , Kursula P . The myelin membrane‐associated enzyme 2',3'‐cyclic nucleotide 3'‐phosphodiesterase: on a highway to structure and function. Neurosci Bull 2014; 30: 956–66 24807122 10.1007/s12264-013-1437-5 PMC5562554

[68] McKerracher L , Rosen KM . MAG, myelin and overcoming growth inhibition in the CNS. Front Mol Neurosci 2015; 8: 51 26441514 10.3389/fnmol.2015.00051 PMC4561339

[69] Pierre K , Pellerin L . Monocarboxylate transporters in the central nervous system: distribution, regulation and function. J Neurochem 2005; 94: 1–14 10.1111/j.1471-4159.2005.03168.x 15953344

[70] Saab AS , Tzvetanova ID , Nave K‐A . The role of myelin and oligodendrocytes in axonal energy metabolism. Curr Opin Neurobiol 2013; 23: 1065–72 24094633 10.1016/j.conb.2013.09.008

[71] Rinholm JE , Hamilton NB , Kessaris N , Richardson WD , Bergersen LH , Attwell D . Regulation of oligodendrocyte development and myelination by glucose and lactate. J Neurosci 2011; 31: 538–48 21228163 10.1523/JNEUROSCI.3516-10.2011 PMC3044866

[72] Lee Y , Morrison BM , Li Y , Lengacher S , Farah MH , Hoffman PN , et al. Oligodendroglia metabolically support axons and contribute to neurodegeneration. Nature 2012; 487: 443–48 22801498 10.1038/nature11314 PMC3408792

[73] Ye F , Chen Y , Hoang T , Montgomery RL , Zhao XH , Bu H , et al. HDAC1 and HDAC2 regulate oligodendrocyte differentiation by disrupting the beta‐catenin‐TCF interaction. Nat Neurosci 2009; 12: 829–38 19503085 10.1038/nn.2333 PMC2701973

[74] Yao ZG , Zhang L , Huang L , Zhu H , Liu Y , Ma CM , et al. Regional and cell‐type specific distribution of HDAC2 in the adult mouse brain. Brain Struct Funct 2013; 218: 563–73 22532304 10.1007/s00429-012-0416-3

[75] Ferrer I . Brain banking In Encyclopedia of the Neurological Sciences 2nd edn, vol. 1 Eds Aminoff MJ, Daroff RB Oxford: Academic Press, 2014; 467–73

[76] Braak H , Braak E . Neuropathological stageing of Alzheimer‐related changes. Acta Neuropathol 1991; 82: 239–59 1759558 10.1007/BF00308809

[77] Barrachina M , Castaño E , Ferrer I . TaqMan PCR assay in the control of RNA normalization in human post‐mortem brain tissue. Neurochem Int 2006; 49: 276–84 16522342 10.1016/j.neuint.2006.01.018

[78] Ferrer I . Diversity of astroglial responses across human neurodegenerative disorders and brain aging. Brain Pathol 2017; 27: 645–74 28804999 10.1111/bpa.12538 PMC8029391

[79] Braak H , Thal DR , Ghebremedhin E , Del Tredici K . Stages of the pathologic process in Alzheimer disease: age categories from 1 to 100 years. J Neuropathol Exp Neurol 2011; 70: 960–69 22002422 10.1097/NEN.0b013e318232a379

[80] Ferrer I . Defining Alzheimer as a common age‐related neurodegenerative process not inevitably leading to dementia. Prog Neurogibol 2012; 97: 38–51 10.1016/j.pneurobio.2012.03.005 22459297

[81] Crary JF , Trojanowski JQ , Schneider JA , Abisambra JF , Abner EL , Alafuzoff I , , et al. Primary age‐related tauopathy (PART): a common pathology associated with human aging. Acta Neuropathol 2014; 128: 755–66 25348064 10.1007/s00401-014-1349-0 PMC4257842

[82] Duyckaerts C , Braak H , Brion JP , et al. PART is part of Alzheimer disease. Acta Neuropathol 2015; 129: 49–756 10.1007/s00401-015-1390-7 PMC4405349 25628035

[83] Yokoi S , Nakano T , Suzuki K . Studies in the white matter of Alzheimer’s disease‐morphological, lipid‐chemical investigation. No To Shinkei 1982; 34: 1197–205 7159549

[84] Soderberg M , Edlund C , Alafuzoff I , Kristensson K , Dallner G . Lipid composition in different regions of the brain in Alzheimer’s disease/senile dementia of Alzheimer’s type. J Neurochem 1992; 59: 1646–53 1402910 10.1111/j.1471-4159.1992.tb10994.x

[85] Svennerholm L , Gottfries CG . Membrane lipids, selectively diminished in Alzheimer brains, suggest synapse loss as a primary event in early‐onset form (type I) and demyelination in late‐onset form (type II*)* . J Neurochem 1994; 62: 1039e1047 8113790 10.1046/j.1471-4159.1994.62031039.x

[86] Roher AE , Weiss N , Kokjohn TA , Kuo YM , Kalback W , Anthony J , et al. Increased Aβ peptides and reduced cholesterol and myelin proteins characterize white matter degeneration in Alzheimer’s disease. Biochemistry 2002; 41: 11080–90 12220172 10.1021/bi026173d

[87] Han X , Holtzman DM , McKeel DW , Kelley J , Morris JC . Substantial sulfatide deficiency and ceramide elevation in very early Alzheimer’s disease: potential role in disease pathogenesis. J Neurochem 2002; 82: 809e818 12358786 10.1046/j.1471-4159.2002.00997.x

[88] Hejazi L , Wong JW , Cheng D , Proschogo N , Ebrahimi D , Garner B , et al. Mass and relative elution time profiling: two‐dimensional analysis of sphingolipids in Alzheimer’s disease brains. Biochem J 2011; 438: 165e175 21639855 10.1042/BJ20110566

[89] Couttas TA , Kain N , Suchowerska AK , Turner N , Fath T , Garner B , et al. Loss of ceramide synthase 2 activity, necessary for myelin biosynthesis, precedes tau pathology in the cortical pathogenesis of Alzheimer’s disease. Neurobiol Aging 2016; 43: 89e100 27255818 10.1016/j.neurobiolaging.2016.03.027

[90] Vlkolinsky R , Cairns N , Fountoulakis M , Lubec G . Decreased brain levels of 2′,3′‐cyclic nucleotide‐3′‐phosphodiesterase in Down syndrome and Alzheimer’s disease. Neurobiol Aging 2001; 22: 547–53 11445254 10.1016/s0197-4580(01)00218-4

[91] Caso F , Agosta F , Filippi M . Insights into white matter damage in Alzheimer's disease: from post‐mortem to in vivo diffusion tensor MRI studies. Neurodegener Dis 2016; 16: 26–33 26618812 10.1159/000441422

[92] Xu J , Chen S , Ahmed SH , Chen H , Ku G , Goldberg MP , et al. Amyloid‐beta peptides are cytotoxic to oligodendrocytes. J Neurosci 2001; 21: Rc118 11150354 10.1523/JNEUROSCI.21-01-j0001.2001 PMC6762453

[93] Lee JT , Xu J , Lee JM , Ku G , Han X , Yang DI , et al. Amyloid beta peptide induces oligodendrocyte death by activating the neutral sphingomyelinase‐ceramide pathway. J Cell Biol 2004; 164: 123–31 14709545 10.1083/jcb.200307017 PMC2171973

[94] Jantaratnotai N , Ryu JK , Kim SU , McLarnon JG . Amyloid beta peptide induced corpus callosum damage and glial activation in vivo. NeuroReport 2003; 14: 1429–33 12960758 10.1097/00001756-200308060-00005

[95] Billings LM , Oddo S , Green KN , McGaugh JL , LaFerla FM . Intraneuronal Aβ causes the onset of early Alzheimer's disease‐related cognitive deficits in transgenic mice. Neuron 2005; 45: 675–88 15748844 10.1016/j.neuron.2005.01.040

[96] Chu TH , Cummins K , Sparling JS , Tsutsui S , Brideau C , Nilsson KPR , et al. Axonal and myelinic pathology in 5xFAD Alzheimer’s mouse spinal cord. PLoS One 2017; 12: e0188218 29176903 10.1371/journal.pone.0188218 PMC5703477

[97] Quintela‐López T , Ortiz‐Sanz C , Serrano‐Regal MP , Gaminde‐Blasco A , Valero J , Baleriola J , et al. Aβ oligomers promote oligodendrocyte differentiation and maturation via integrin β1 and Fyn kinase signaling. Cell Death Dis 2019; 10: 445 31171765 10.1038/s41419-019-1636-8 PMC6554322

[98] Roseborough A , Ramirez J , Black SE , Edwards JD . Associations between amyloid β and white matter hyperintensities: a systematic review. Alzheimers Dement 2017; 13: 1154–67 28322203 10.1016/j.jalz.2017.01.026

[99] Collins‐Praino LE , Francis YI , Griffith EY , Wiegman AF , Urbach J , Lawton A , et al. Soluble amyloid beta levels are elevated in the white matter of Alzheimer’s patients, independent of cortical plaque severity. Acta Neuropathol Commun 2014; 2: 83 25129614 10.1186/s40478-014-0083-0 PMC4147157

[100] McAleese KE , Firbank M , Dey M , Colloby SJ , Walker L , Johnson M , et al. Cortical tau load is associated with white matter hyperintensities. Acta Neuropathol Commun 2015; 3: 60 26419828 10.1186/s40478-015-0240-0 PMC4589169

[101] McAleese KE , Walker L , Graham S , Moya ELJ , Johnson M , Erskine D , et al. Parietal white matter lesions in Alzheimer’s disease are associated with cortical neurodegenerative pathology, but not with small vessel disease. Acta Neuropathol 2017; 134: 459–73 28638989 10.1007/s00401-017-1738-2 PMC5563333

[102] Stokin GB , Lillo C , Falzone TL , Brusch RG , Rockenstein E , Mount SL , et al. Axonopathy and transport deficits early in the pathogenesis of Alzheimer’s disease. Science 2005; 307: 1282–88 15731448 10.1126/science.1105681

[103] Sahara N , Perez PD , Lin WL , Dickson DW , Ren Y , Zeng H , et al. Age‐related decline in white matter integrity in a mouse model of tauopathy: an *in vivo* diffusion tensor magnetic resonance imaging study. Neurobiol Aging 2014; 35: 1364e1374 24411290 10.1016/j.neurobiolaging.2013.12.009 PMC4729397

[104] Gagyi E , Kormos B , Castellanos KJ , Valyi‐Nagy K , Korneff D , LoPresti P , et al. Decreased oligodendrocyte nuclear diameter in Alzheimer’s disease and Lewy body dementia. Brain Pathol 2012; 22: 803–10 22429607 10.1111/j.1750-3639.2012.00595.x PMC4181948

[105] Behrendt G , Baer K , Buffo A , Curtis MA , Faull RL , Rees MI , et al. Dynamic changes in myelin aberrations and oligodendrocyte generation in chronic amyloidosis in mice and men. Glia 2013; 61: 273–86 23090919 10.1002/glia.22432

[106] Nielsen HM , Ek D , Avdic U , Orbjorn C , Hansson O , Netherlands Brain B ; Veerhuis R , et al. NG2 cells, a new trail for Alzheimer’s disease mechanisms? Acta Neuropathol Commun 2013; 1: 7 24252600 10.1186/2051-5960-1-7 PMC4046664

[107] Al‐Mashhadi S , Simpson JE , Heath PR , Dickman M , Forster G , Matthews FE , et al. Oxidative glial cell damage associated with white matter lesions in the aging human brain. Brain Pathol 2015; 25: 565–74 25311358 10.1111/bpa.12216 PMC4861214

[108] Tse KH , Herrup K . DNA damage in the oligodendrocyte lineage and its role in brain aging. Mech Ageing Dev 2017; 161: 37–50 27235538 10.1016/j.mad.2016.05.006 PMC5124419

[109] Rivera A , Vanzuli I , Arellano JJ , Butt A . Decreased regenerative capacity of oligodendrocyte progenitor cells (NG2‐Glia) in the ageing brain: a vicious cycle of synaptic dysfunction, myelin loss and neuronal disruption? Curr Alzheimer Res 2016; 13: 413–18 26567743 10.2174/1567205013666151116125518

[110] Butt AM , Chacon de la Rocha I , Rivera A . Oligodendroglial cells in Alzheimer’s disease In Neuroglia in Neurodegenerative Diseases. Eds Verkhratsky A, Ho MS, Zorec R, Parpura. V Singapore: Springer Nature, 2019, 325–34.

[111] Pak K , Chan SL , Mattson MP . Presenilin‐1 mutation sensitizes oligodendrocytes to glutamate and amyloid toxicities, and exacerbates white matter damage and memory impairment in mice. NeuroMolecular Med 2003; 3: 53–64 12665676 10.1385/NMM:3:1:53

[112] Desai MK , Sudol KL , Janelsins MC , Mastrangelo MA , Frazer ME , Bowers WJ . Triple‐transgenic Alzheimer's disease mice exhibit region‐specific abnormalities in brain myelination patterns prior to appearance of amyloid and tau pathology. Glia 2009; 57: 54–65 18661556 10.1002/glia.20734 PMC2584762

[113] Desai MK , Mastrangelo MA , Ryan DA , Sudol KL , Narrow WC , Bowers WJ . Early oligodendrocyte/myelin pathology in Alzheimer’s disease mice constitutes a novel therapeutic target. Am J Pathol 2010; 177: 1422–35 20696774 10.2353/ajpath.2010.100087 PMC2928974

[114] Dong XX , Zhang HY , Li HY , Liu PH , Sui Y , Sun XH . Association between Alzheimer’s disease pathogenesis and early demyelination and oligodendrocyte dysfunction. Neural Regen Res 2018; 13: 908–14 29863022 10.4103/1673-5374.232486 PMC5998637

[115] Gu L , Wu D , Tang X , Qi X , Li X , Bai F , et al. Myelin changes at early stage of 5xFAD nice. Brain Res Bull 2018; 137: 285–293 29288735 10.1016/j.brainresbull.2017.12.013

[116] Kamphuis W , Orre M , Kooijman L , Dahmen M , Hol EM . Differential cell proliferation in the cortex of the APPswePS1dE9 Alzheimer's disease mouse model. Glia 2012; 60: 615–29 22262260 10.1002/glia.22295

[117] Ferrer I , Bella R , Serrano MT , Martí E , Guionnet N . Arteriolosclerotic leucoencephalopathy in the elderly and its relation to white matter lesions in Binswanger's disease, multi‐infarct encephalopathy and Alzheimer's disease. J Neurol Sci 1990; 98: 37–50 2230830 10.1016/0022-510x(90)90180-u

[118] Moody DM , Bell MA , Challa VR . Features of the cerebral vascular pattern that predict vulnerability to perfusion or oxygenation deficiency: an anatomic study. Am J Neuroradiol 1990; 11: 431–39 2112304 PMC8367475

[119] Brown WR , Moody DM , Challa VR , Thore CR , Anstrom JA . Venous collagenosis and arteriolar tortuosity in leukoaraiosis. J Neurol Sci 2002; 203–204: 159–63 10.1016/s0022-510x(02)00283-6 12417376

[120] Grinberg LT , Thal DR . Vascular pathology in the aged human brain. Acta Neuropathol 2010; 119: 277–290 20155424 10.1007/s00401-010-0652-7 PMC2831184

[121] Brown WR , Thore CR . Review: cerebral microvascular pathology in ageing and neurodegeneration. Neuropathol Appl Neurobiol 2011; 37: 56–74 20946471 10.1111/j.1365-2990.2010.01139.x PMC3020267

[122] Bridges LR , Andoh J , Lawrence AJ , Khoong CHL , Poon W , Esiri MM , et al. Blood‐brain dysfunction and cerebral small vessel disease (arteriolosclerosis) in brains of older people. J Neuropathol Exp Neurol 2014; 73: 1026–33 25289893 10.1097/NEN.0000000000000124 PMC4209852

[123] Liua H , Yanga Y , Xiaa Y , Zhua W , Leakd RK , Weia Z , et al. Aging of cerebral white matter. Ageing Res Rev 2017; 34: 64–76 27865980 10.1016/j.arr.2016.11.006 PMC5250573

[124] Kalaria RN , Premkumar DR , Pax AB , Cohen DL , Lieberburg I . Production and increased detection of amyloid beta protein and amyloidogenic fragments in brain microvessels, meningeal vessels and choroids plexus in Alzheimer's disease. Mol Brain Res 1996; 35: 58–68 8717340 10.1016/0169-328x(95)00180-z

[125] Thal DR , Griffin WS , de Vos RA , Ghebremedhin E . Cerebral amyloid angiopathy and its relationship to Alzheimer’s disease. Acta Neuropathol 2008; 115: 599–609 18369648 10.1007/s00401-008-0366-2

[126] Silverberg GD , Messier AA , Miller MC , Machan JT , Majmudar SS , Stopa EG , , et al. Amyloid efflux transporter expression at the blood‐brain barrier declines in normal aging. J Neuropathol Exp Neurol 2010; 69: 1034–1043 20838242 10.1097/NEN.0b013e3181f46e25

[127] Silverberg GD , Miller MC , Messier AA , Majmudar S , Machan JT , Donahue JE , et al. Amyloid deposition and influx transporter expression at the blood‐brain barrier increase in normal aging. J Neuropathol Exp Neurol 2010; 69: 98–108 20010299 10.1097/NEN.0b013e3181c8ad2f

[128] Kalaria RN , Pax AB . Increased collagen content of cerebral microvessels in Alzheimer's disease. Brain Res 1995; 707: 349–52 10.1016/0006-8993(95)01250-8 8821769

[129] Farkas E , Luiten PGM . Cerebral microvascular pathology in aging and Alzheimer's disease. Progr Neurobiol 2001; 64: 575–611 10.1016/s0301-0082(00)00068-x 11311463

[130] Grammas P , Yamada M , Zlokovic B . The cerebromicrovasculature: a key player in the pathogenesis of Alzheimer's disease. J Alzheimers Dis 2002; 4: 217–23 12226540 10.3233/jad-2002-4311

[131] Ervin JF , Pannell C , Szymansky M , Welsh‐Bohmer K , Schmechel DE , Hulette CM . Vascular smooth muscle actin is reduced in Alzheimer disease brain: a quantitative analysis. J Neuropathol Exp Neurol 2004; 63: 735–41 15290898 10.1093/jnen/63.7.735

[132] Perez E , Barrachina M , Rodriguez A , Torrejón‐Escribano B , Boada M , Hernandez I , et al. Aquaporin expression in the cerebral cortex is increased at early stages of Alzheimer's disease. Brain Res 2007; 1128: 164–74 17123487 10.1016/j.brainres.2006.09.109

[133] Anderson VC , Lenar DP , Quinn JF , Rooney WD . The blood‐brain barrier and microvascular water exchange in Alzheimer's disease. Cardiovasc Psychiatry Neurol 2011; 2011: 615829 21687589 10.1155/2011/615829 PMC3114411

[134] Erickson MA , Banks WA . Blood–brain barrier dysfunction as a cause and consequence of Alzheimer's disease. J Cereb Blood Flow Metab 2013; 33: 1500–13 23921899 10.1038/jcbfm.2013.135 PMC3790938

[135] Zenaro E , Piacentino G , Constantin G . The blood‐brain barrier in Alzheimer's disease. Neurobiol Dis 2017; 107: 41–56 27425887 10.1016/j.nbd.2016.07.007 PMC5600438

[136] Gallart‐Palau X , Serra A , Hase Y , Tan CF , Chen CP , Kalaria RN , et al. Brain‐derived and circulating vesicle profiles indicate neurovascular unit dysfunction in early Alzheimer’s disease. Brain Pathol 2019; 29: 593–605 30629763 10.1111/bpa.12699 PMC8028379

[137] Scheibel AB , Duong TH , Jacobs R . Alzheimer's disease as a capillary dementia. Ann Med 1989; 21: 103–7 2669844 10.3109/07853898909149194

[138] De la Torre JC . Alzheimer's disease is a vasocognopathy: a new term to describe its nature. Neurol Res 2004; 26: 517–24 15265269 10.1179/016164104225016254

[139] Kalback W , Esh C , Castano EM , Rahman A , Kojohn T , Luehrs DC , et al. Atherosclerosis, vascular amyloidosis and brain hypoperfusion in the pathogenesis of sporadic Alzheimer's disease. Neurol Res 2004; 26: 525–9 15265270 10.1179/016164104225017668

[140] Bartzokis G . Alzheimer’s disease as homeostatic responses to age‐related myelin breakdown. Neurobiol Aging 2011; 32: 1341–71 19775776 10.1016/j.neurobiolaging.2009.08.007 PMC3128664

[141] Cai Z , Xiao M . Oligodendrocytes and Alzheimer's disease. Int J Neurosci 2016; 126: 97–104 26000818 10.3109/00207454.2015.1025778

[142] Nasrabady SE , Rizvi B , Goldman JE , Brickman AM . White matter changes in Alzheimer’s disease: a focus on myelin and oligodendrocytes. Acta Neuropathol Commun 2018; 6: 22 29499767 10.1186/s40478-018-0515-3 PMC5834839

[143] Mot AI , Depp C , Nave KA . An emerging role of dysfunctional axon‐oligodendrocyte coupling in neurodegenerative diseases. Dialogues Clin Neurosci 2018; 20: 283–92 30936768 10.31887/dcns.2018.20.4/amot PMC6436955

[144] Ferrer I . Oligodendrogliopathy in neurodegenerative diseases with abnormal protein aggregates: the forgotten partner. Prog Neurogibol 2018; 169: 24–54 10.1016/j.pneurobio.2018.07.004 30077775

